# Automatic EEG artifact detection using a local-global feature fusion network in time and time-frequency domains

**DOI:** 10.1186/s42494-026-00248-4

**Published:** 2026-03-01

**Authors:** Qindong Yu, Chuan Lin, Weibo Wang, Zhanhui Feng, Ming Wen

**Affiliations:** 1 School of Electrical Engineering, Southwest Jiaotong University, Xi’an road 999, Chengdu, 611756 Sichuan China; 2 School of Electrical and Electronic Information, Xihua University, Hongguang avenue 999, Chengdu, 610039 Sichuan China; 3 Department of Neurology, Guizhou Provincial People’s Hospital, Zhongshan road east 83, Guiyang, 550002 Guizhou China; 4 Department of Neurology, the Third People’s Hospital of Chengdu, Qinglong street 82, Chengdu, 610014 Sichuan China

**Keywords:** Signal processing, EEG artifact detection, Deep learning model, Multi-domain feature fusion, Temporal-lobe-epilepsy

## Abstract

**Background:**

Video electroencephalographies (VEEGs) are often affected by artifacts, which can diminish clinicians’ efficiency in interpreting VEEG data and potentially result in diagnostic errors. Conversely, certain ictal VEEG artifacts caused by automatisms, such as rhythmic chewing and blinking, may offer significant diagnostic clues for temporal lobe epilepsy. To address the challenges mentioned above, this study aims to develop an algorithm capable of automatically identifying and classifying artifact types, assisting clinicians in distinguishing interference signals from seizure signals.

**Methods:**

This paper proposes a Local-Global Feature Fusion Network based on Time-Domain and Time-Frequency Domain (LG-TDTFD-Net) for detecting and classifying temporal lobe epilepsy artifacts. The model processes time-domain and time-frequency signals separately, leveraging convolutional neural network (CNN) to extract local features from both domains. Additionally, convolution operations are performed on the features extracted by the Transformer to capture deep local features, resulting in a feature set that simultaneously encompasses global, deep, and shallow local information.

**Results:**

Experimental results demonstrate that the proposed method excels in detecting and classifying temporal lobe epilepsy artifacts, outperforming existing baseline models on clinical and public datasets.

**Conclusions:**

The method effectively exploits the complementarity among the three feature types by utilizing feature concatenation and CNN-based fusion, enhancing feature representation and improving model generalization.

**Trial registration:**

The Chinese Clinical Trial Registry, ChiCTR2300079319. Registered 30 December 2023, https://www.chictr.org.cn/showproj.html?proj=214123.

## Background

Epilepsy is one of the neurological disorders associated with the disruption of brain activity that affects approximately 50 million people around the world [1]. Temporal lobe epilepsy is the most common type of focal epilepsy diagnosed in tertiary medical centers (approximately 66%) and is also the most prevalent type of epilepsy undergoing surgical intervention in clinical practice. Video Electroencephalography (VEEG) is widely used to detect changes in brain electrical activity to diagnose epilepsy [2, 3]. However, because of the weak nature of EEG signals, they are highly susceptible to interference from artifacts. These artifacts can be broadly categorized into physiological artifacts, such as electrooculogram (EOG), electromyogram (EMG), and non-physiological artifacts, including environmental noise and electrode malfunctions [4]. During long-term EEG monitoring, artifact signals can easily be confused with physiological waveforms, leading to misinterpretations by clinicians. In previous studies, most of the researchers focused on the removal of artifacts. Regression models [5, 6] as well as blind source separation methods, such as independent component analysis (ICA) [7–9] are commonly used. Deep learning methods have also achieved impressive results in this task [10]. Artifact removal helps restore EEG signal features and reduces the interference of artifacts in EEG detection.

**Fig. 1:**
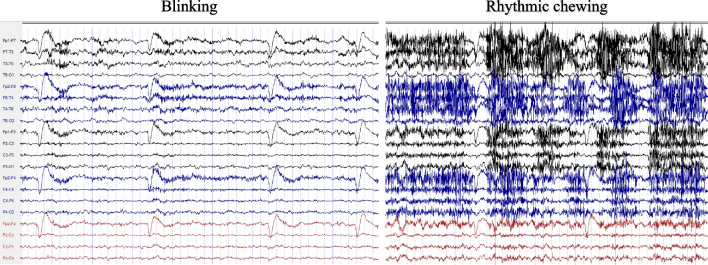
Automatism artifacts. During temporal lobe epilepsy seizures, rhythmic blinking and chewing artifacts may manifest as two types of pseudo-interference

However, we found that it can also provide valuable diagnostic insights [11]. For example, in cases of temporal lobe epilepsy [12, 13], seizures originating from the temporal lobe are frequently accompanied by artifacts caused by automatisms such as rhythmic chewing and blinking, as illustrated in Fig. 1, which are easily confused with the normal type of chewing and eye movement. Manual detection of artifacts in long-term VEEG recordings remains inefficient and prone to errors, especially when the patient might be in a blind spot of video surveillance during a seizure. Using artificial intelligence for VEEG analysis can effectively identify and classify artifacts, reducing the workload of clinicians and improving diagnostic accuracy. Hence, developing an algorithm capable of automatically detecting and classifying artifacts holds significant potential value for clinical diagnostics.

Previous studies have used numerous traditional machine learning methods to classify EEG artifact signals. These methods typically involve manually extracting features and feeding them into classifiers. For example, support vector machines (SVM) have been used for the binary classification of eye movement artifacts and normal signals [14]. Linear logistic regression was used to distinguish five types of artifacts [15]. A 0.5s sliding window was used to extract features, and a K Nearest Neighbor (KNN) classifier was employed to detect artifacts, which were then sent to an Long Short-Term Memory (LSTM) network for artifact removal [16]. However, most traditional feature extraction methods are task-dependent and require manual intervention to extract features. In addition, these approaches face challenges in feature selection and optimization of classifier parameters, which can limit their effectiveness and adaptability.

Subsequently, researchers began exploring deep learning approaches, including models based on convolutional neural networks (CNN) [17, 18]. CNN can automatically learn and extract important time-dependent features through sliding convolutional kernels, making them highly effective for time-series signal feature extraction tasks. Applying CNNs to compare classification performance before and after artifact removal [19]. CNN was used to detect different types of artifacts in multi-channel EEG signals [20]. However, due to the limited size of kernels, CNNs primarily capture local receptive field features, which makes them incapable of capturing long-term dependencies that are critical for time-series analysis. To address the problem of long-term dependencies, RNNs and LSTMs were proposed [21–23]. However, their sequential nature hinders parallel training.

Recently, attention-based transformer models have made significant strides in natural language processing and image analysis due to their ability to capture global dependencies [24]. Leveraging long-term temporal relationships, Transformers have also demonstrated promising performance in EEG analysis. In EEG studies, global information plays a crucial role; however, relying solely on Transformers for feature extraction often leads to neglecting local signal characteristics. For instance, Signals were segmented with a 25% overlap and used a transformer to extract features from each segment, followed by feature selection and classification with a CatBoost classifier [25]. Using CNN to extract local features along the temporal and spatial dimensions of multi-lead EEG signals before feeding them into a Transformer [26]. Relying solely on Transformers for feature extraction often leads to neglecting local signal characteristics. Simultaneously, input the spectrogram into both the transformer and CNN for feature extraction [27].

Existing methods often failed to adequately balance or effectively integrate global and local feature extraction, resulting in incomplete feature representations. Additionally, these approaches typically focus on either time-domain or frequency-domain features, leading to a limited diversity of information and incomplete feature sets. The previous study primarily focused on classifying non-pathological artifacts, without distinguishing diagnostically relevant artifacts that could aid clinical interpretation. Our study specifically differentiates between easily confused normal eye movement, chewing, and their ictal counterparts (blinking and rhythmic chewing during epileptic seizures).

To overcome the previously identified limitations, a novel method called the Local-Global Feature Fusion Network Based on Time-Domain and Time-Frequency Domain (LG-TDTFD-Net) is presented. This approach employs dual inputs: time-domain signals and time-frequency spectrum, which are processed separately by Time-domain feature extraction (TDFE) and Time-Frequency-domain feature extraction (TFDFE) modules. This dual-input framework allows the network to learn features from the time domain while simultaneously capturing the temporal variations in frequency, thereby enhancing the overall comprehensiveness of the extracted information. Within the TDFE and TFDFE modules, initial feature extraction is performed using CNNs, followed by processing through a Transformer. The Transformer excels in identifying and capturing global relationships and long-term dependencies within the input sequences, effectively addressing the limitations of CNNs regarding global feature extraction. Additionally, we implement a further convolution operation on the features extracted by the Transformer. This step intends to capture deep local features, resulting in a feature set that merges global attributes with deep and shallow local information. This fused feature set significantly enhances the model’s generalization capabilities, enabling a more comprehensive understanding and accurate processing of complex and diverse temporal signals.

The contributions are summarized as follows:

- A new CNN-transformer-based classification network, named Local-Global Feature Fusion Network Based on Time-Domain and Time-Frequency-Domain is proposed in this paper for EEG artifact classification, which includes pathological artifacts unlike traditional interfering artifact types. The proposed LG-TDTFD-Net outperformed other baseline models, demonstrating better effectiveness.
- In the neural network architecture, shallow and deep local, and global features are extracted at different levels. These three levels of features were then fused to enrich the overall feature representation. This approach enhances the model’s discriminative power and feature extraction capabilities and improves its adaptability and robustness to complex data.
- By integrating the time-domain and time-frequency domain features, the model captures both time-dependent features and temporal variations in frequency, forming a comprehensive feature set. This enhances the capability to identify one-dimensional signals.

## Methods

### Overview

**Fig. 2:**
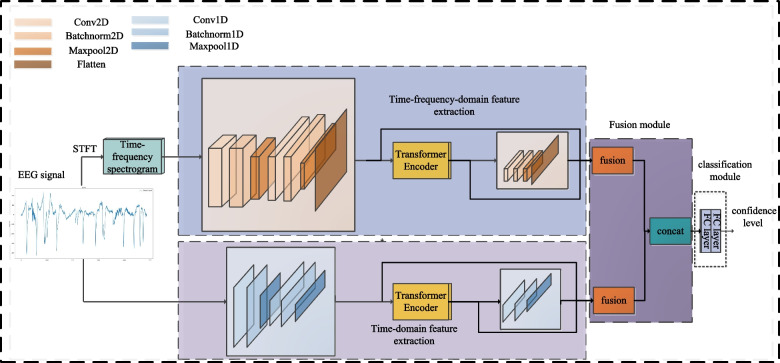
The overall architecture: The entire model is divided into two branches: one extracts time-domain features and the other extracts time-frequency-domain features, which are then fused at the end. Different colors in the diagram represent different modules, as shown in the top left corner

In the proposed model, LG-TDTFD-Net adopts a dual-branch architecture that simultaneously processes the time-domain signal and its time–frequency spectrogram. These are handled by the TDFE module and the TFDFE module, respectively. The overall structure of the model is shown in Fig. 2.

TDFE is performed by *CONV*1*Da*, which extracts the local features ${F_{CONV1Da}}$ from the time-domain signal. These features were then input into a transformer module to learn the global features, resulting in ${F_{trans1}}$. Next, *CONV*1*Db* is used to extract the local features ${F_{CONV1Db}}$ from ${F_{trans1}}$, which are fused with *CONV*1*Da* and ${F_{trans1}}$ to form the time-domain feature set. Meanwhile, TFDFE uses a short-time fourier transform (STFT) to generate the time-frequency spectrogram of the 1D signal. *CONV*2*Da* was then applied to extract features, which were flattened to obtain ${F_{CONV2Da}}$. These features were input into the transformer to learn the global features, resulting in ${F_{trans2}}$. Then, *CONV*2*Db* extracts local features ${F_{CONV2Db}}$ from ${F_{trans2}}$, which are fused with ${F_{CONV2Da}}$ and ${F_{trans2}}$ to form the time-frequency domain feature set. Finally, the time-domain and time-frequency domain features are combined to form a comprehensive feature set, enhancing the generalization ability of the model.

### Time-domain feature extraction

**Fig. 3:**
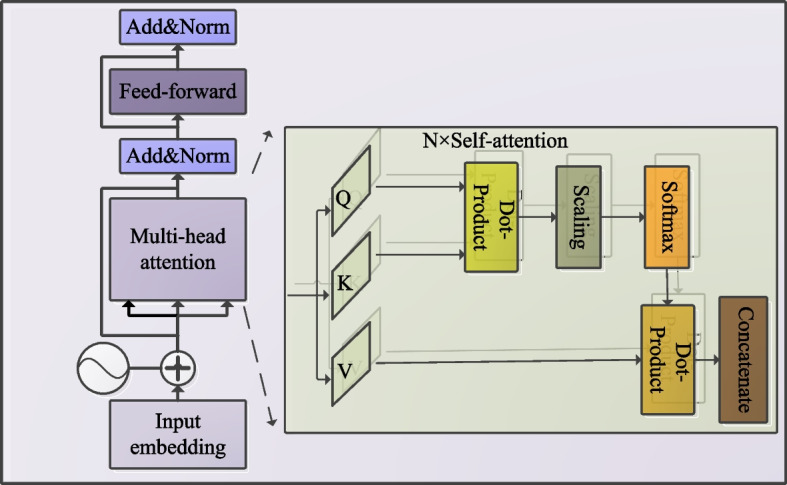
Time-domain feature extraction module

This module extracts shallow local features, deep local features, and global features from the one-dimensional time-domain signal, and then concatenates and fuses them to form the time-domain feature set. The structure of the module is shown in Fig. 3.

The input signal is *x(n)* in a one-dimensional convolutional network. After two convolutional layers, the first layer uses a larger convolution kernel to cover a wider temporal range, which helps to capture global relationships. The second layer, which has a smaller convolution kernel, focuses on rapid signal changes and local features. The model effectively integrates features across different time scales by combining the operations of both large and small kernels. This approach is beneficial for analyzing complex signals with varying frequency components, such as brainwaves or heartbeats in physiological signals, enabling a more comprehensive representation and differentiation of the signal characteristics. To enhance the training process and mitigate overfitting, batch normalization was applied after each convolutional layer. An Exponential Linear Unit (ELU) was used as the nonlinear activation function, and a max-pooling layer was applied afterward. The pooling layer smooths time-based features, preventing overfitting and reducing computational complexity. The module’s parameters are shown in Table 1: The feature sequence ${F_{CONV1Da}}$, denoted as ${F_{CONV1Da}} \in {R^{T {\times } n}}$, serves as the input to the Transformer, where n represents the embedding dimension and T is the sequence length. *CONV*1*Da* represents the entire one-dimensional convolution module:

1 $$\begin{aligned} {F_{CONV1Da}} = CONV1Da(x(n)) \end{aligned}$$

**Table 1:** 1D-CNN parameters

Module	Input	Output	Kernel	Stride
CONV1d	1	16	8	4
BatchNorm1d	16	16	/	/
Maxpooling1d	16	16	4	2
CONV1d	16	40	4	2
BatchNorm1d	40	40	/	/
Maxpooling1d	40	40	2	1

Including two convolutional layers, two normalization layers, and two pooling layers

**Fig. 4:**
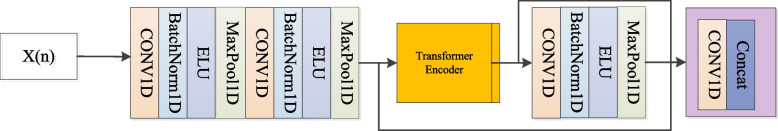
Transformer encoder

The Transformer comprises multiple layers of self-attention mechanisms, as depicted in Fig. 4. The self-attention mechanism enables global modeling of long-range and short-range temporal dependencies within the input sequence, thereby capturing complex dynamic patterns in the signals more effectively. The multi-head attention mechanism enhances this process by executing several self-attention computations in parallel, each in a different subspace, and then concatenating the outputs. This approach allows the model to capture richer feature relationships across diverse dimensions, further boosting its capacity for representation and modeling, as formalized in the following equation:

2 $$\begin{aligned} Attention(Q,K,V) = soft\max (\frac{{Q{K^T}}}{{\sqrt{{d_k}} }})V \end{aligned}$$

3 $$\begin{aligned} MultiHead(Q,K,V) = Concat(hea{d_1},...,hea{d_h}) \end{aligned}$$

4 $$\begin{aligned} hea{d_l} = Attention({Q_l},{K_l},{V_l}) \end{aligned}$$

The query matrix Q, key matrix K, and value matrix V are the core components of the self-attention mechanism. The mechanism computes the similarity between the query and key vectors using dot-product calculations to obtain attention scores. These scores were then used to weight the input sequence elements. The essence of the self-attention mechanism lies in computing the relevance between each query vector and all key vectors. Typically, after calculating the similarity using the dot-product, a scaling factor $\sqrt{{d_k}}$ is applied, where ${d_k}$ is the dimensionality of the key vectors (is equal to the sequence length), which stabilizes the numerical behavior and prevents dot products from becoming excessively large when ${d_k}$ is high. In this study, the Transformer encoder is composed of 6 layers, each equipped with 5 attention heads and an embedding size of 40. The feature sequence ${F_{CONV1Da}}$ is processed through the transformer encoder to produce ${F_{trans1}} \in {R^{T{\times }n}}$, which retains the same sequence length as ${F_{CONV1Da}}$. Subsequently, ${F_{trans1}}$ is passed through a 1D convolutional layer, to extract its local features, resulting in ${F_{CONV1Db}} \in {R^{{T_1}{\times }n}}$, where *n* represents the embedding dimension and ${T_1}$ is the sequence length.

5 $$\begin{aligned} {F_{trans1}} = transformer({F_{CONV1Da}}) \end{aligned}$$

6 $$\begin{aligned} {F_{CONV1Db}} = CONV1Db({F_{trans1}}) \end{aligned}$$

This step aims to capture deeper-level local features, enriching the feature set by complementing the already extracted global and shallow local features. Combining global dependencies modeled by the transformer and the detailed local patterns identified by *CONV*1*Db* enhances the representation’s capacity, enabling a more comprehensive and robust understanding of the input signals.

### Time-frequency-domain feature extraction

**Fig. 5:**
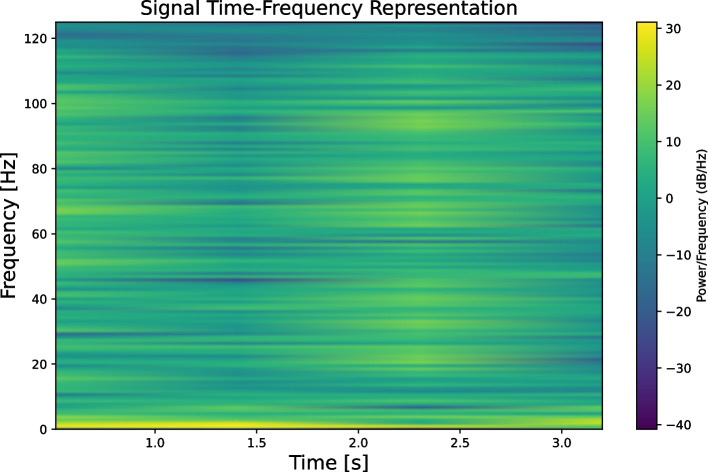
Time-frequency-domain feature extraction

This module takes the spectrogram of the one-dimensional signal as input, extracts its local and global features, and then concatenates and fuses them to form the time–frequency domain feature set. The architecture is illustrated in Fig. 5.

**Fig. 6:**
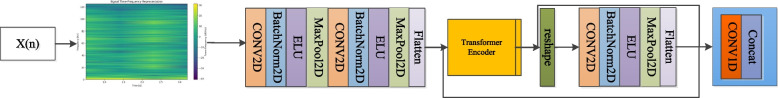
Time-frequency spectrum

A one-dimensional signal is transformed into a time-frequency spectrogram using the STFT [28], where the horizontal axis represents time and the vertical axis represents frequency. This spectrogram illustrates how the frequency components vary over time, as shown in Fig. 6. We applied STFT using a Hamming window of length 250 and an overlap of 200 to balance time and frequency resolution. Longer windows improve frequency resolution, while higher overlaps enhance spectrogram smoothness but increase computational cost. The selected parameters offer a practical trade-off between representation quality and efficiency, making them suitable for our EEG data and model requirements. Inspired by [29, 30], a spectrogram was used as the input for the two-layer 2D CNN. The first convolutional layer, with a kernel size of (3,1), extracts features along the frequency axis, whereas the second convolutional layer, with a kernel size of (1,3), extracts features along the time axis. Additionally, normalization, activation, and pooling operations were performed. This design aimed to capture frequency-domain features and extract features representing frequency variations over time. This ensures that the extracted features retain temporal characteristics, facilitating the subsequent input into the transformer. The parameters of the 2D CNN module are presented in Table 2.

**Table 2:** 2D-CNN parameters

Module	Input	Output	Kernel	Stride
CONV2d	1	16	(1,3)	1
BatchNorm2d	16	16	/	/
Maxpooling2d	16	16	(2,2)	1
CONV2d	16	40	(3,1)	1
BatchNorm2d	40	40	/	/
Maxpooling2d	40	40	(2,2)	1

Including two convolutional layers, two normalization layers, and two pooling layers

The matrix ${F_{CONV2Da}}\in {R^{n{\times }Hout{\times }Wout}}$ represents the flattened 2D convolution features extracted from the spectrogram by the *CONV*2*Da* module, where *n* represents the number of output channels, and *Hout* and *Wout* are the height and width of the feature map, respectively. This 3D feature matrix is then flattened into a 2D feature matrix ${F_{CONV2Da}} \in {R^{n{\times }\left( {H{out} \cdot Wout} \right) }}$.

7 $$\begin{aligned} {F_{CONV2Da}} = Flatten(CONV2Da(spectrum)) \end{aligned}$$

To prepare the features for input into the Transformer, the matrix is transposed into the format required by the Transformer, resulting in a feature sequence of shape ${F_{CONV2Da}} \in {R^{\left( {Hout{\cdot }Wout} \right) {\times }n}}$. This transformation ensures that the feature sequence has a temporal-like structure that facilitates the ability of the transformer to capture global frequency variations across the feature space. ${F_{CONV2Da}}$ is then input to the transformer encoder, which undergoes encoding to produce ${F_{trans2}} \in {R^{\left( {Hout{\cdot }Wout} \right) {\times }n}}$. After encoding, ${F_{trans2}}$ is reshaped back into a 3D matrix and passed through a 2D convolutional layer, to extract deeper local features called ${F_{CONV2Db}} \in {R^{{T_2}{\times }n}}$, where *n* represents the embedding dimension and ${T_2}$ is the sequence length, as formalized in the following equation:

8 $$\begin{aligned} {F_{trans1}} = transformer({F_{CONV1Da}}) \end{aligned}$$

9 $$\begin{aligned} {F_{CONV2Db}} = CONV2Db({F_{trans2}}) \end{aligned}$$

This step focuses on capturing intricate and deep local patterns within the spectrogram, enriching the representation with highly detailed frequency-domain information.

### Time-domain and time-frequency-domain feature fusion

The Feature Fusion module combines the features extracted from both the time domain and time–frequency domain to form a complete and unified feature set.

Three levels of features are extracted from both the time and frequency domains, resulting in the time-domain feature ${F_{td}} \in {R^{{T_3}{\times }n}}$ and the time-frequency-domain feature ${F_{tfd}} \in {R^{{T_4}{\times }n}}$, where *n* represents the embedding dimension and ${T_3},{T_4}$ are the sequence length:

- Shallow local features (${F_{CONV1Da}}$, ${F_{CONV2Da}}$): capture the fine-grained details and low-level patterns in the data, such as edges and textures, which are critical for identifying basic local information.
- Global features(${F_{trans1}}$, ${F_{trans2}}$): capture long-range dependencies and global contextual information, providing an overall understanding of the data structure.
- Deep local features(${F_{CONV1Db}}$,${F_{CONV2Db}}$): Extracts higher-level complex patterns and advanced semantic information, enabling a more abstract feature representation.

These features were concatenated to form a comprehensive feature set for both the time and frequency domains. The concatenated features were fused for each domain using a CNN with a kernel size of 1, as represented in the equations, to enhance the expressiveness and robustness of the model. Combining these three information levels significantly improves the feature extraction capability and generalization ability of the model. The CNN further optimizes this fusion process by compressing and extracting richer information while increasing the adaptability and stability of the model for complex data.

10 $$\begin{aligned} \begin{array}{l} {F_{td}} = CONV1d(cancatenate({F_{CONV1Da}}\\ + {F_{trans1}} + {F_{CONV1Db}})) \end{array} \end{aligned}$$

11 $$\begin{aligned} \begin{array}{l} {F_{tfd}} = CONV1d(cancatenate({F_{CONV2Da}}\\ + {F_{trans2}} + {F_{CONV2Db}})) \end{array} \end{aligned}$$

Finally, the features from both the time and time-frequency domains are concatenated to form a complete feature set $F \in {R^{{{\left( {{T_3} + T} \right) }_4}{\times }n}}$, as illustrated in the equations.

12 $$\begin{aligned} F = concatenate({F_{td}} + {F_{tfd}}) \end{aligned}$$

**Fig. 7:**
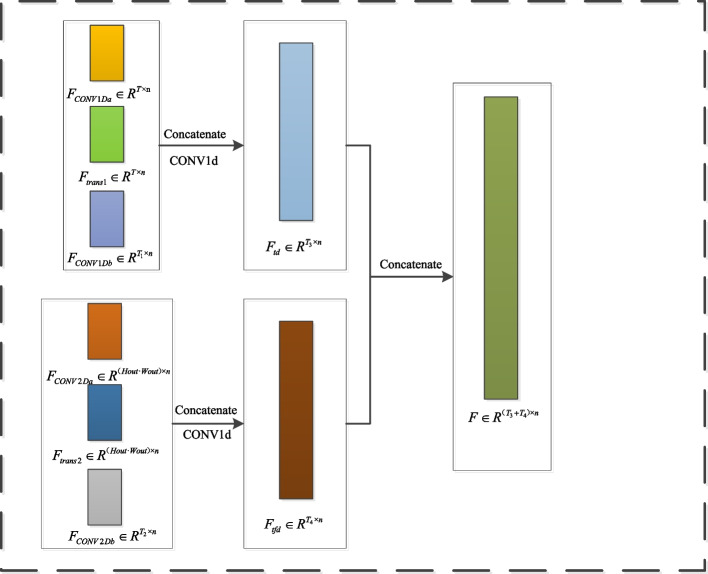
The concatenate module

This ensures that the resulting feature set encapsulates temporal and spectral information, allowing the model to understand both time-dependent changes and frequency variations over time, ultimately enhancing robustness and effectiveness. As shown in Fig. 7, the overall architecture demonstrates the integration and processing steps required to achieve comprehensive and robust feature extraction and fusion.

### Classification module

The resulting total feature set was passed through two fully connected layers, reducing the dimensions to m, where m is the number of classes. Then, the softmax function was applied to output the class probabilities. The class was determined based on the confidence level. Given a real-valued $z \in {R^K}$, the i-th element of the softmax function is calculated as follows:

13 $$\begin{aligned} soft\max ({z_i}) = \frac{{{e^{{z_i}}}}}{{\sum \nolimits _{J = 1}^K {{e^{zj}}} }} \end{aligned}$$

${z_i}$ is the i-th element of vector z, and the denominator is the sum of the exponentials of all the elements in z, ensuring that the output probabilities sum to 1. Cross-entropy is used as the loss function:

14 $$\begin{aligned} L(y, \hat{y}) = -\sum y_i \log (\hat{y_i}) \end{aligned}$$

y is the true label distribution, typically a one-hot vector, where the index corresponding to the correct class is 1, and all other positions are 0. *hat* *y* is the predicted probability distribution from the model, obtained using the softmax function. The Adam optimizer was used for parameter updates to ensure the stability and efficiency of the training process.

## Results

### Dataset

**Fig. 8:**
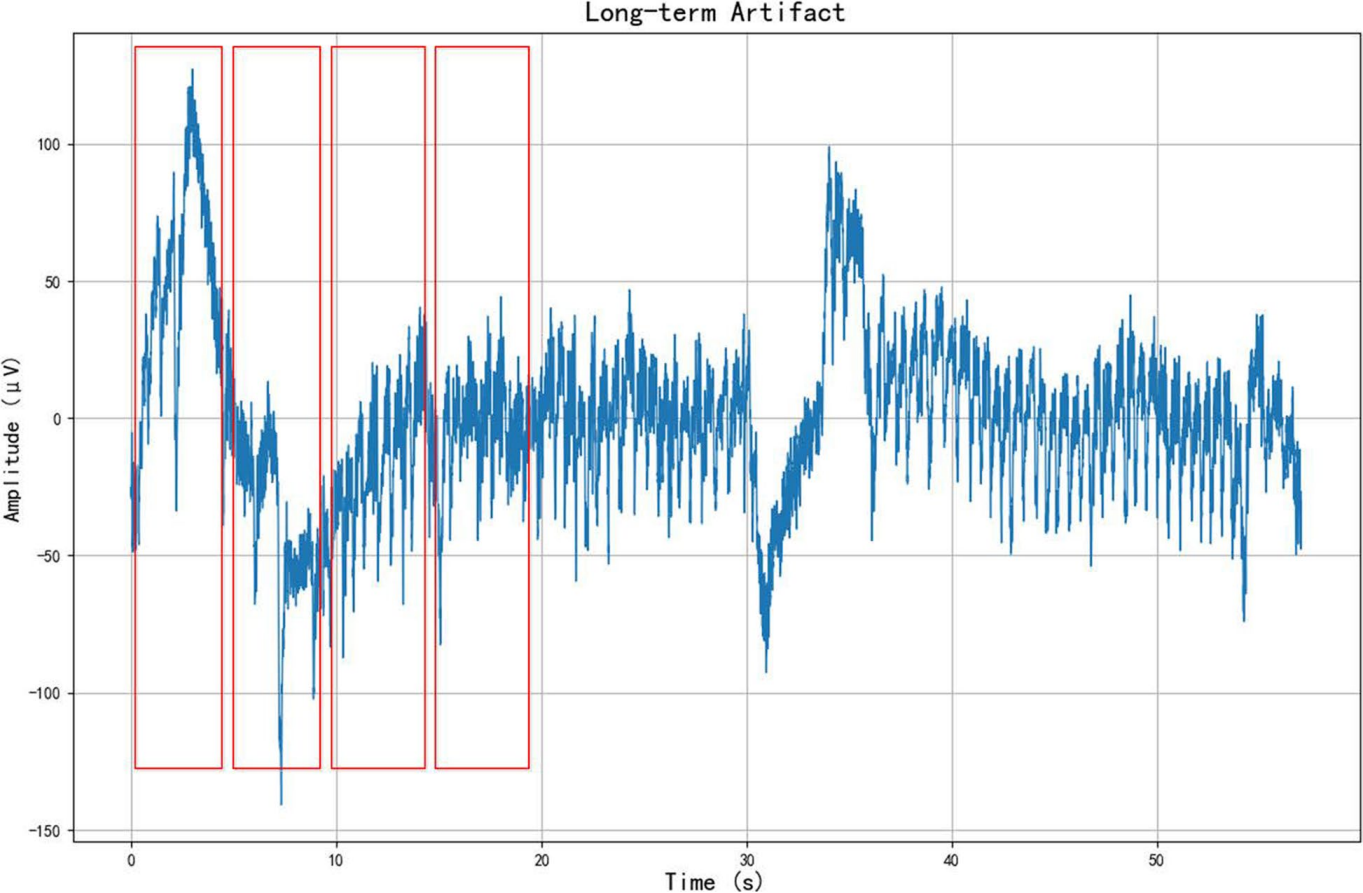
Data segment. Extract non-overlapped short segments from a long artifact signal

**Fig. 9:**
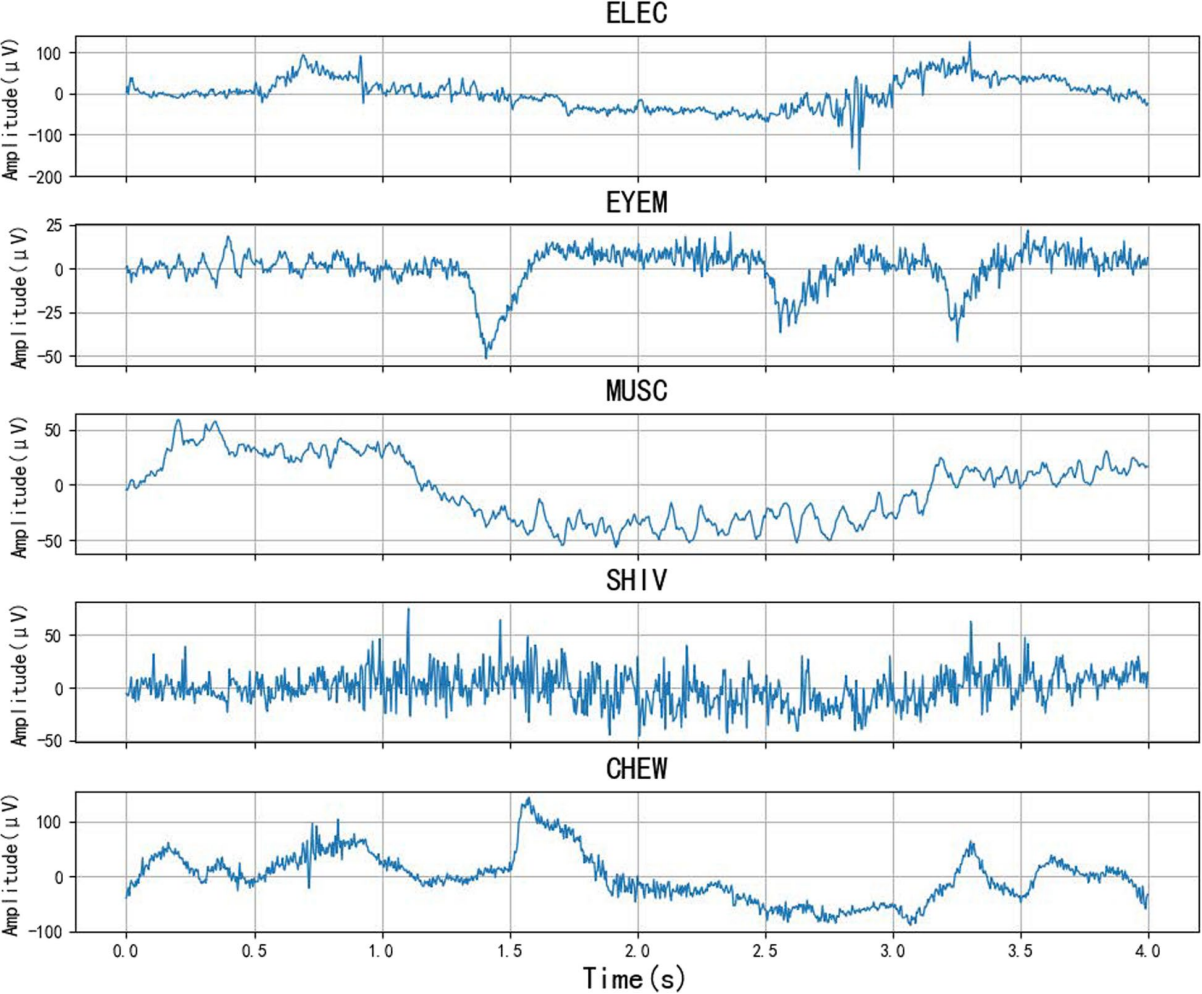
Public dataset

This study used two datasets: 1) a publicly available EEG dataset provided by the Temple University Hospital (TUH) of Philadelphia (Pennsylvania) [31], the Temple University Artifact Corpus (TUAR) (public dataset). The data were pre-processed and re-referenced to a bipolar montage for analysis. 2) The clinical dataset from the Third People’s Hospital of Chengdu (private dataset)was also preprocessed and re-referenced to a bipolar montage:

The Temple University dataset includes 310 recordings, totaling 99.98 hours, each meticulously annotated to identify artifacts. Annotations within the corpus are classified into five categories: eye movement (EYEM), muscle (MUSC), chewing (CHEW), electrode (ELEC), and shiver (SHIV) artifacts. First, we extracted the segments where artifacts occurred from the entire file based on the annotations. We segmented the annotated data and selected the segments with a sampling frequency of 250 Hz. Labeled segments with durations of 4 s or longer were divided into 1 s, 2 s, 4 s, and 5 s segments, as shown in Fig. 8. This approach aims to compare the performance of models with different segment lengths. The specific data distributions are presented in Table 3. To eliminate the influence of the data volume on performance, the number of data segments was kept consistent across different segment lengths. The collected data are shown in Fig. 9.

**Table 3:** Number of each artifact in the public dataset

Label	CHEW	MUSC	ELEC	EYEM	SHIV
Number	1169	1678	1127	1476	872

The quantity of segments of different lengths is the same

**Fig. 10:**
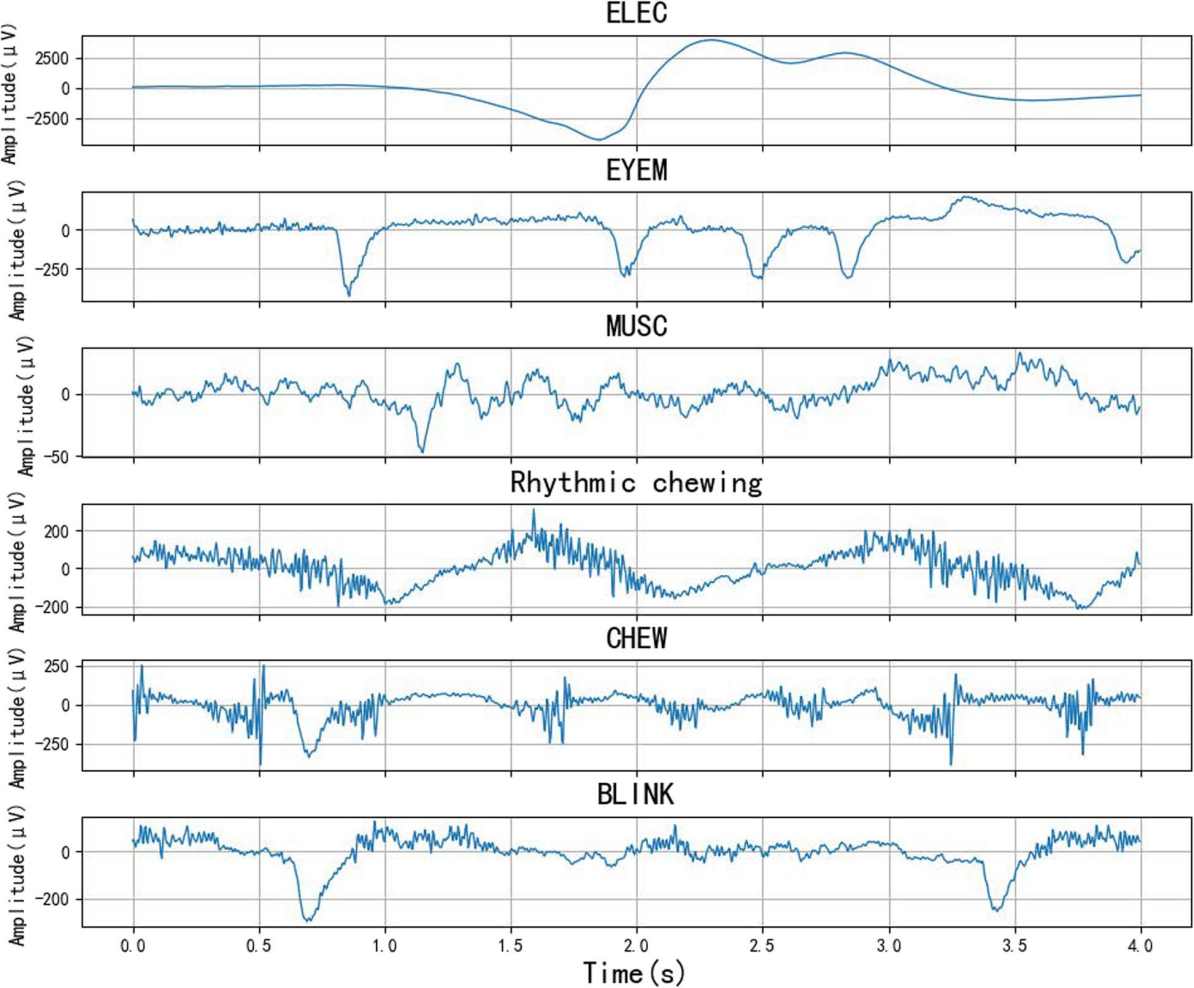
Private dataset

The private dataset consisted of 23 VEEG recordings obtained from individuals with temporal lobe epilepsy in our dataset, each lasting 1 h, for a total of 23h. The dataset was annotated by experienced neurologists from the Third People’s Hospital of Chengdu. This study aimed to investigate artifacts during episodes of temporal lobe epilepsy. Therefore, we extracted datasets of muscle artifacts, eye movements, chewing, electrode artifacts, blinking (BLINK), and rhythmic chewing (RC) from the frontal-temporal and frontal regions (FP1-F7, FP2-F8, FP1-F3, FP2-F4 leads). Our data did not include shiver artifacts. Several pre-processing steps are required because the dataset consists of raw clinical data. The private dataset has a sampling frequency of 256 Hz. To align with the TUAR, we downsampled the data to 250 Hz. Since the sampling rate conversion factor is not an integer, direct downsampling is not feasible. Therefore, a resampling approach is adopted. First, a low-pass filter is applied to remove components above the target Nyquist frequency (125 Hz). Then, the resampling parameters are calculated to determine the upsampling factor (125) and the downsampling factor (128), ensuring that 256/250 = 128/125. The signal is then upsampled by a factor of 125 (by inserting zeros), followed by convolution with an anti-aliasing filter to smooth the interpolated signal. Finally, the filtered signal is downsampled by a factor of 128 (uniform decimation) to obtain a signal with a sampling rate of 250 Hz. A Butterworth filter was applied for bandpass filtering in the 0.5–80Hz range, followed by notch filtering to remove the 50 Hz power line interference. Finally, the data were segmented into 4 s segments as the public dataset. Examples of private datasets are shown in Fig. 10, and the number of each artifact is listed in Table 4.

**Table 4:** Number of each artifact in the private dataset

Label	CHEW	MUSC	ELEC	EYEM	RC	BLINK
Number	400	400	400	400	400	400

### Evaluation metrics

In this study, we employed three widely used classification metrics to evaluate the artifact detection performance of the proposed model: accuracy, recall, and F1-score. The F1-score serves as a comprehensive metric by harmonizing precision and recall, addressing potential imbalances in the class distribution. These metrics are derived from the confusion matrix and are formally defined as follows:

15 $$\begin{aligned} Accuracy = \frac{{TP+TN}}{{TP +TN+ FP + FN}} \end{aligned}$$

16 $$\begin{aligned} Recall = \frac{{TP}}{{TP + FN}} \end{aligned}$$

17 $$\begin{aligned} F1-score = \frac{{2TP}}{{2TP + FP + FN}} \end{aligned}$$

where* TP *(True Positives) and *TN *(True Negatives) correspond to the counts of correctly identified positive and negative samples, respectively. Meanwhile, *FP*(False Positives) and *FN*(False Negatives) represent the counts of incorrectly classified positive and negative samples, respectively.

### Training process

The experiment was conducted using Python 3.11 in PyCharm and trained on a single GeForce 3090 GPU. Our model was set up with the Adam optimizer with a learning rate of 0.0002, $\beta 1$=0.9,$\beta 2$=0.999, and a batch size of 200. The dataset was divided into training and testing sets at an 8:2 ratio, and a five-fold cross-validation strategy was employed. We obtained the accuracy, recall, and F1-score, and calculated their average values.

### Selection of best segment length

This study experimented with four different segment lengths: 1 s, 2 s, 4 s, and 5 s to determine the most suitable for our model. We compared the accuracy, recall, and F1-score for each segment length, and we bold the highest evaluation metric for each type of artifact. The results are presented in Table 5.

**Table 5:** Different segment comparison of accuracy, recall, and F1-score

Label	Length	Accuracy(%)	F1-score	Recall
CHEW	1s	83.51%	0.904	0.835
	2s	90.40%	0.940	0.904
	4s	**99.36%**	**0.989**	**0.994**
	5s	92.72%	0.946	0.927
MUSC	1s	97.16%	0.971	0.972
	2s	**97.19%**	**0.972**	**0.972**
	4s	93.75%	0.932	0.938
	5s	91.13%	0.913	0.911
EYEM	1s	71.48%	0.765	0.715
	2s	91.91%	0.921	0.919
	4s	**93.87%**	**0.942**	**0.939**
	5s	84.21%	0.848	0.842
ELEC	1s	63.89%	0.685	0.639
	2s	61.47%	0.672	0.615
	4s	85.02%	0.850	0.850
	5s	**87.4%**	**0.853**	**0.874**
SHIV	1s	83.33%	0.924	0.833
	2s	94.44%	0.971	0.944
	4s	**100%**	**1**	**1**
	5s	98.7%	0.987	0.987
AVG	1s	85.2%	0.876	0.852
	2s	87.2%	0.896	0.872
	4s	**94.1%**	**0.943**	**0.941**
	5s	90.6%	0.908	0.906

Bold text indicates the best result

Bold text indicates the best result.

Based on the experiments, it was found that the 4 s segments yielded the highest overall accuracy, F1-score, and recall for the model. The highest accuracy for most labels was also achieved with the 4 s segments, particularly with significant improvements in detecting CHEW artifacts. This is likely due to the larger amount of information contained in the 4-second data segments; shorter segments might not fully capture some artifacts, leading to insufficient information. However, in detecting MUSC artifacts, the 4 s segments were slightly less effective than the 2 s segments, and in detecting ELEC artifacts, the 4 s segments were slightly less effective than the 5 s segments.

Among the tested segment lengths, the 4-second segment yielded the highest performance across multiple evaluation metrics, including accuracy, F1-score, and recall. This indicates that 4-second segments capture richer temporal information and provide a more discriminative input, enabling the model to more effectively learn both local and global features.

Although increasing the segment length leads to a proportional growth in model parameters (from 4.5M for 1-second segments to 23M for 5-second segments), the corresponding training time per epoch increases only moderately (from 3.5s to 7.1s), as illustrated in Table 6.

**Table 6:** Comparison of computational efficiency across different segment lengths

Length	Training time	Parameter
1s	3.5s	4.5M
2s	4.8s	9.2M
4s	6.2s	18M
5s	7.1s	23M

Notably, while shorter segments (1s and 2 s) result in smaller models, they also suffer from a clear drop in classification performance. In contrast, although the 5-second segment increases the parameter count, it fails to yield further improvement beyond the 4-second case. Therefore, considering both model performance and training efficiency, we selected the 4-second segment as the optimal configuration for subsequent experiments.

### Ablation study

To comprehensively validate the effectiveness of each module in our model, we designed a series of ablation experiments to gradually reveal the contribution of each component to the performance of the model.

1. **CNN**: Only the CNN model is used to extract local features and directly input them into the fully connected layer.
2. **CNN-Transformer**: In this model, the CNN extracts features from the 1D signal, which is then input into a transformer model to obtain the transformer feature sequence.
3. **CNN-Transformer-fusion**: This model integrates the local features from the CNN and global features from the transformer.
4. **CNN-Transformer-CNN-fusion**: Based on method (2), we further applied a 1D convolution to extract features from the Transformer output sequence. These features were then concatenated with the CNN’s local features and the Transformer’s global features, followed by another 1D convolution for final fusion.

The detailed results of our experiments are presented in Table 7. After adding the Transformer module, the accuracy, F1-score, and recall increased by 6.7%, 5.8%, and 6.7%, respectively. The addition of the fusion module further improved these metrics by 3%, 1%, and 3%, respectively. Introducing deep local features led to additional gains of 3%, 5%, and 3%. while incorporating time–frequency domain features with time domain features resulted in further improvements of 3.1%, 2.3%, and 3.1%, respectively.

**Table 7:** Accuracy, F1-score, recall of ablation study

Method	Accuracy	F1-score	Recall
CNN	0.783	0.802	0.783
CNN-Transformer	0.85	0.86	0.85
CNN-Transformer-fusion	0.88	0.87	0.88
CNN-Transformer-CNN-fusion	0.91	0.92	0.91
LG-TDTFD-Net	0.941	0.943	0.941

**Fig. 11:**
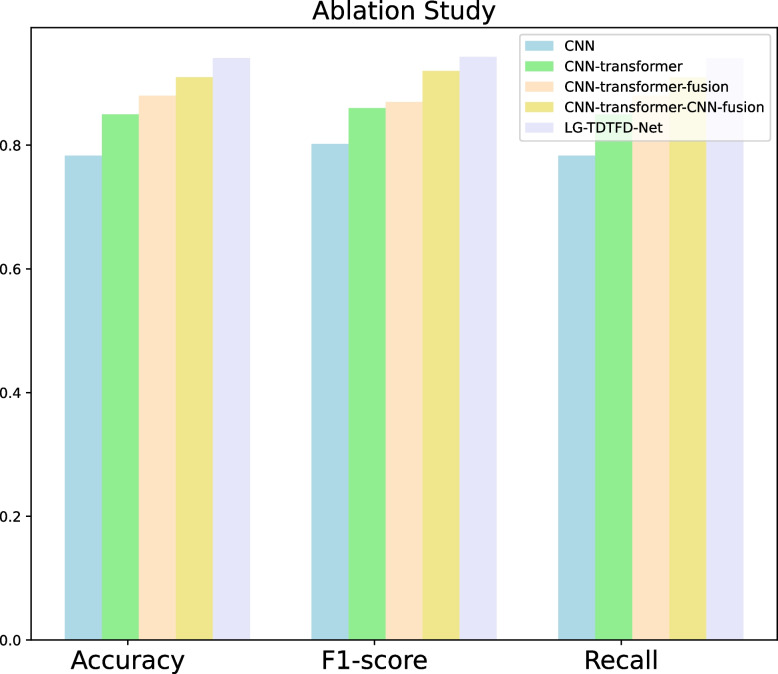
Accuracy, F1-score and recall of ablation study

Adding each module raises all metrics, and our model shows improvements in all metrics compared with the other models, validating the effectiveness of our approach. Figure 11 visually reflects the model’s improvement.

**Fig. 12:**
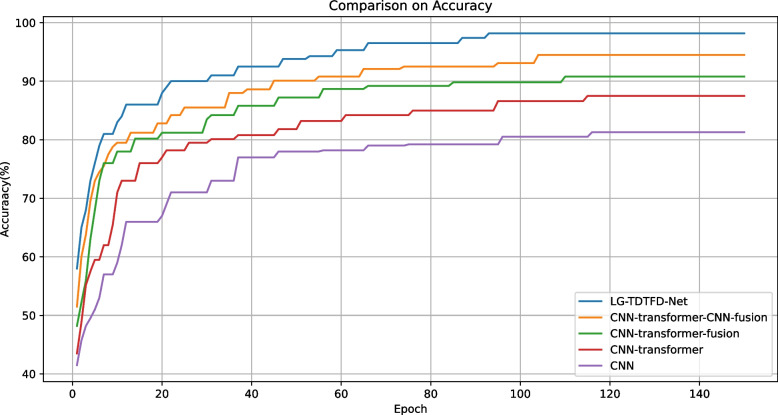
Accuracy convergence comparison of ablation study

**Fig. 13:**
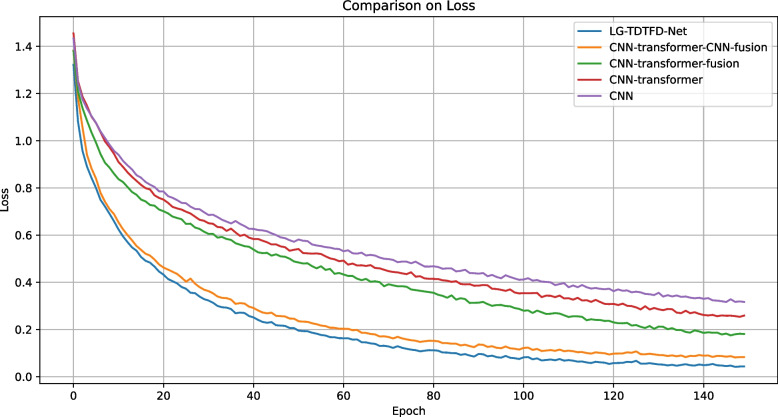
Training loss of ablation study

As shown in Figs. 12 and 13, the addition of each module led to improvements in both accuracy and training loss, along with faster convergence. The proposed LG-TDTFD-Net achieved higher accuracy, lower training loss, and faster convergence, demonstrating superior training efficiency and effectiveness.

### Fusion strategy comparison

To evaluate the effectiveness of different feature fusion strategies, we conducted two additional experiments:

1. **Feature-weighted-fusion**: This method employs an MLP-based attention mechanism to dynamically assign weights to shallow, deep local, and global features before fusing them.
2. **Feature-weighted-CNN-fusion**: Building on Feature-weighted-fusion, this method further introduces a CNN layer to enhance interactions between the different feature types.

The results are presented in Table 8. The Feature-weighted fusion method showed a 4% decrease across all evaluation metrics compared to the proposed CNN-Transformer-CNN fusion approach. Although the Feature-weighted-CNN fusion achieved similar accuracy and recall, its F1-score was 2% lower. Therefore, we adopted the CNN-transformer-CNN-fusion in our final model.

**Table 8:** Comparison of different fusion strategies

Method	Accuracy	F1-score	Recall
CNN-Transformer-CNN-fusion	0.91	0.92	0.91
Feature-weighted-fusion	0.87	0.88	0.87
Feature-weighted-CNN-fusion	0.91	0.90	0.90

### Baseline comparison

To validate the superiority of our model, we compared it to other baseline models. However, because relatively few methods have been applied to this dataset, we selected some of the latest EEG epilepsy classification models for comparison:

1. CNN-tra [25]: This approach inputs a window and slices it into 0.5-second overlapping segments with a 25% overlap. It extracts features from the segments and classifies them using CatBoost.
2. CNN-LSTM [23]: This model uses a two-layer 1D CNN with 64 output channels, a kernel size of 3, and a stride of 1, followed by an LSTM with a kernel size of 50.
3. ResNet-LSTM [32]: This approach first extracts features using 1D convolution, and then inputs them separately into ResNet and LSTM. The features are fused and passed into a fully connected layer for classification.
4. RCNN-LSTM [33]: This model extracts temporal features from the signal using LSTM, extracting time-frequency features using a CNN from spectrograms. The two types of features were fused and input into a fully connected layer.
5. PDBFusNet [27]: In this work, the EEG signal is extracted by Mel Frequency Cepstral Coefficients (MFCC). The extracted features are then delivered to the parallel dual branches to simultaneously capture the short- and long-term dependencies of the EEG signal.

As shown in Table 9, we bold the two highest accuracy rates for each type of artifact. Compared to other baseline models, LG-TDTFD-Net demonstrates significant improvements in accuracy: for CHEW artifacts, it outperforms the second-best PDBFusnet by 0.36%; for EYEM artifacts, it surpasses CNN-Tra by 8.17%; for ELEC artifacts, it exceeds the second-best RCNN-LSTM by 2.26%; and for MUSC artifacts, it outperforms RCNN-LSTM by 1.2%.

**Table 9:** Baseline model performance comparison of accuracy

Method	Accuracy				
	CHEW	ELEC	EYEM	MUSC	SHIV
CNN-tra	94.7%	73.5%	**85.7%**	82.6%	65.5%
CNN-LSTM	85.30%	71.01%	84.05%	73.30%	98.74%
Resnet-LSTM	66.13%	80.17%	69.62%	84.77%	85.96%
RCNN-LSTM	83.87%	**82.76%**	77.22%	**92.55%**	**100%**
PDBFusNet	**99.03%**	69.36%	83.34%	83.55%	97.73%
LG-TDTFD-Net	**99.36%**	**85.02%**	**93.87%**	**93.75%**	**100%**

Bold text indicates the best result

### Private dataset experiment

To validate the generalization capability of our model, we evaluated it on a private dataset using a model trained on a public dataset and compared its performance with that of other baseline models. Due to the absence of shiver artifacts in the private dataset, we conducted testing using only four types of artifacts: MUSC, EYEM, ELEC, and CHEW, with 400 samples for each artifact type. Our model achieved excellent results for this task, with accuracies of CHEW 98.25%, ELEC 83.75%, EYEM 91.75%, and MUSC 88.75%. Except for MUSC, where the accuracy was slightly lower than that of RCNN-LSTM, our model achieved the highest accuracy across all artifact categories, demonstrating its robust performance and strong generalization capability. We bold the highest accuracy for each artifact. The detailed results are presented in Table 10.

**Table 10:** Test results of baseline models on the private dataset

Method	Accuracy			
	CHEW	ELEC	EYEM	MUSC
CNN-LSTM	82.00%	71.25%	80.50%	71.75%
Resnet-LSTM	61.75%	79.25%	62.75%	80.75%
RCNN-LSTM	80.75%	80.25%	75.75%	**89.25%**
PDBFusNet	92.50%	67.75%	81.25%	81.00%
LG-TDTFD-Net	**98.25%**	**83.75%**	**91.75%**	88.75%

Bold text indicates the best result

Having validated our model’s performance and generalization capability on the public dataset, we applied it to the objective of our current study, which is to distinguish between artifacts during seizures and those during non-seizure periods. In this classification task, we obtained the following results in Table 11:

**Table 11:** Accuracy, F1-score, recall of private dataset

Label	Accuracy	F1-score	Recall
CHEW	97.5%	0.969	0.975
MUSC	96.3%	0.957	0.963
EYEM	93.8%	0.950	0.938
RC	97.5%	0.969	0.975
ELEC	96.3%	0.963	0.963
BLINK	95.0%	0.956	0.950
AVG	96.0%	0.961	0.960

We conducted classification on six types of artifacts: CHEW, MUSC, EYEM, ELEC, RC, and BLINK. We achieved an average accuracy rate of 96.0%, an F1-score of 0.961, and a recall rate of 0.960.

## Discussion

This study proposes a novel model, LG-TDTFD-Net, which integrates CNN and Transformer architectures to jointly analyze both time-domain and time–frequency domain features. The aim is to comprehensively capture the intrinsic patterns in different types of artifact signals, enabling effective identification of epileptic-related information hidden within artifacts while filtering out irrelevant noise.

In ablation studies, compared with traditional CNN models, the introduction of Transformer modules provides self-attention mechanisms capable of capturing long-range dependencies within sequences. This enhances global connectivity among features and improves the model’s understanding of sequential data. The accuracy, F1-score, and recall increased by 6.7%, 5.8%, and 6.7%, respectively. Further comparisons reveal that the CNN-Transformer-Fusion structure, which integrates both local and global features, significantly enhances the model’s ability to represent complex patterns, resulting in improvements of 3%, 1%, and 3% in accuracy, F1-score, and recall, respectively. Building upon this, although the Transformer captures global features and contextual relationships, it induces a smoothing effect that weakens local features when processing signal details. The CNN-Transformer-CNN-Fusion model further addresses this by extracting deep local features. These deep local features leverage convolutional kernels with local receptive fields to further mine and refine the global features, enhancing local temporal details, thereby improving the discriminative power of the features. The complementarity of these three types of features improves the model’s discriminative power, feature extraction capabilities, and generalization performance, though it leads to improvements of 3%, 5%, and 3% in accuracy, F1-score, and recall, respectively. Compared with CNN-Transformer-CNN-Fusion, the proposed LG-TDTFD-Net introduces a time–frequency feature extraction module. Through spectral analysis, it reveals the dynamic frequency changes of the signal over time, allowing the model to simultaneously learn critical features from both time and time–frequency domains. This leads to improved robustness and generalization, and leads to improvements of 3.1%, 2.1%, and 3.1% in accuracy, F1-score, and recall, respectively.

In the fusion strategy comparison experiment, experimental results show that our proposed fusion strategy outperforms the other two methods, achieving an accuracy of 91%, F1-score of 0.92, and recall of 0.91. By concatenating shallow, deep local, and global features followed by a CNN layer, our method enables effective interaction and integration of complementary information across feature types. In contrast, the Feature-weighted fusion method, although capable of dynamically adjusting feature importance, struggles to capture deep interactions between features. The Feature-weighted-CNN-fusion approach builds upon this by introducing a CNN-based fusion module to enhance the interactons between features, similar to the CNN-Transformer-CNN-fusion structure, leading to noticeable improvements in evaluation metrics. This further confirms the effectiveness of using CNNs to facilitate feature interaction. Overall, our method not only achieves better performance but also maintains a simpler architecture with lower computational cost, demonstrating its practicality and advantage in multi-feature fusion tasks.

Baseline experiment results demonstrate that, compared with existing methods that rely solely on either time-domain or time–frequency domain features, LG-TDTFD-Net achieves superior performance when handling non-stationary EEG signals. On the public dataset, the model achieved an accuracy of 94.1%, a recall of 0.941, and an F1-score of 0.943. In the private dataset, it performed even better, with an accuracy of 96.0%, a recall of 0.960, and an F1-score of 0.961. These results not only validate the model’s effectiveness but also indicate its strong adaptability across both public and private datasets, consistently outperforming baseline models on multiple evaluation metrics.

For example, models such as CNN-LSTM [23], ResNet-LSTM [32], and RCNN-LSTM [33] incorporate LSTM to enhance temporal modeling capabilities. Among them, RCNN-LSTM combines time and frequency domain feature extraction, outperforming the others and further validating the effectiveness of incorporating time–frequency information for classification. However, RCNN-LSTM mainly focuses on local features, whereas LG-TDTFD-Net captures both local and global features, further improving its ability to recognize complex EEG. PDBFusNet is a parallel dual-input network that uses CNN and Transformer to extract local and global features from Mel Frequency Cepstral Coefficients (MFCC), respectively, followed by feature fusion. However, this network lacks the extraction of time-domain features. Moreover, the CNN-Tra [25] model, similar to traditional machine learning approaches, first extracts features and outputs softmax probabilities for different segments, which are then used as inputs to a classifier. This pipeline heavily relies on preselected features and may lead to underutilization of information. In contrast, LG-TDTFD-Net mitigates this limitation with its end-to-end dual-domain fusion architecture.

To evaluate generalization capability, we tested models trained on the public dataset using the private dataset. The results demonstrate that our proposed model generalizes better than other baseline models. Additionally, we conducted a six-class classification experiment based on the private dataset. Unlike the public dataset, the private dataset includes two additional artifact types: RC and BLINK, which originate from automatisms and can serve as important indicators for identifying temporal lobe epilepsy seizures. The experiment achieved promising results, providing a meaningful reference for future clinical validation.

The superior performance of LG-TDTFD-Net is mainly attributed to the advantages of its dual-domain fusion framework:

1. Time-domain features are effective in capturing transient changes and waveform morphology, while time–frequency features reveal non-stationary properties such as frequency changes. The parallel extraction and integration of the models of both types of features provide a more comprehensive representation of the signal.
2. CNN excels at capturing local spatial patterns, whereas the Transformer’s self-attention mechanism enables modeling of long-range temporal dependencies. Their combination allows the model to accurately identify the spatio-temporal dynamics associated with epileptic activity.
3. The ability to robustly process non-stationary signals across varying noise levels and data sources ensures stable model performance, which is critical for clinical applications.

Despite the significant progress achieved, this study still faces several limitations and challenges. Due to current constraints, the proposed method has not yet been implemented or tested in real clinical scenarios and remains at the stage of laboratory evaluation and methodological refinement. The lack of standardized interfaces among EEG devices from different manufacturers poses challenges for model deployment, as additional adaptation is often required. Furthermore, most commercial EEG analysis systems are closed platforms, making it difficult to directly integrate external deep learning models. Additionally, the current study covers only a restricted range of artifact types, limiting the model’s applicability in diverse clinical scenarios. The model is trained and validated exclusively on temporal lobe epilepsy data. Since epilepsy originating in other brain regions exhibits distinct EEG characteristics, the transferability of the current model remains unverified. This work primarily focuses on identifying epileptic-relevant features within artifacts, but does not address artifact removal, which is equally crucial for obtaining clean EEG signals for downstream analysis.

Future research will focus on expanding the model’s capabilities in artifact detection and removal to encompass a wider range of clinically relevant artifact types. In addition, efforts will be made to improve the generalization of the model by extending its applicability to various forms of epilepsy originating in different regions of the brain. For future clinical deployment, collaboration with device manufacturers could be considered to develop standardized data interfaces, which would enhance the model’s generalizability and compatibility. Additionally, the model can be packaged as a callable module and integrated into existing clinical systems via an intermediate platform, minimizing disruption to current system architecture. Finally, integrating the model into clinical decision support systems will be a key direction, aiming to provide meaningful assistance to clinicians, reduce diagnostic workload, and improve overall efficiency.

## Conclusions

This study successfully developed and validated an innovative CNN-Transformer model, LG-TDTFD-Net, for artifact detection in EEG signals associated with temporal lobe epilepsy. The core innovation of the model lies in its dual-domain (time and time–frequency) parallel processing architecture, which enables more comprehensive and robust capture of the intrinsic characteristics of EEG signals. This design allows for effective differentiation of artifacts and accurate identification of key information relevant to seizure detection.

Experiments on both public and private datasets demonstrated excellent results (Public dataset: Accuracy = 94.1%, Recall = 0.941, F1-score = 0.943; Private dataset: Accuracy = 96.0%, Recall = 0.960, F1-score = 0.961), fully validating the model’s effectiveness, robustness, and superior generalization ability. Notably, on the public dataset, the model outperformed baseline models across multiple evaluation metrics, highlighting the significant advantage of the dual-domain fusion strategy in processing complex nonstationary EEG signals.

In summary, LG-TDTFD-Net shows great potential as a clinical decision support tool. It can assist physicians in more efficiently and accurately interpreting EEG data with artifacts, focusing on information truly associated with epileptic activity, thereby improving diagnostic efficiency and reliability. Although the current study is limited by the range of artifact types and epilepsy origins covered, and focuses on artifact detection rather than removal, it lays a solid foundation for future work. Future research will aim to detect a broader spectrum of artifacts and expand the model’s applicability.

## Data Availability

All data generated or analyzed in this study are included in this manuscript.

## Abbreviations

CNN: Convolutional Neural Networks
KNN: K Nearest Neighbor
LG-TDTFD-Net: Local-Global Feature Fusion Network Based on Time-Domain and Time-Frequency Domain
LSTM: Long Short-Term Memory
STFT: Short-Time Fourier Transform
TUAR: Temple University Artifact Corpus
VEEG: Video Electroencephalography
